# Understanding Role of DNA Repair and Cytochrome p-450 Gene Polymorphisms in Cervical Cancer Patient Treated With Concomitant Chemoradiation

**DOI:** 10.3389/bjbs.2021.10120

**Published:** 2022-02-24

**Authors:** Mohammad Abbas, Vandana Singh Kushwaha, Kirti Srivastava, Monisha Banerjee

**Affiliations:** ^1^ Molecular and Human Genetics Laboratory, Department of Zoology, University of Lucknow, Lucknow, India; ^2^ Department of Personalized and Molecular Medicine, Era University, Lucknow, India; ^3^ Department of Radiotherapy, All India Institute of Medical Sciences, Nagpur, India; ^4^ Department of Radiotherapy, King George’s Medical University, Lucknow, India

**Keywords:** cervical cancer, CYP1A1, CYP2E1, DNA repair gene, polymorphisms, treatment outcome

## Abstract

**Background:** Evidences suggest that single nucleotide polymorphisms (SNPs) can be considered as potential biomarkers for disease progression and therapeutic response in cervical cancer. The present study investigated the association of *CYP1A1* T>C (rs4646903), *CYP1A1* A>G (rs1048943), *CYP2E1* T>A (rs6413432), *RAD51* G>C (rs1801320), *XRCC1* G>A (rs25487), *XRCC2* G>A (rs3218536) and *XRCC3* C>T (rs861539) polymorphisms with treatment outcome of cisplatin based chemoradiation (CRT).

**Methods:** Total 227 cervical cancer cases, treated with the same chemoradiotherapy regimen were selected for the study. Genotyping analysis was performed by PCR-restriction fragment length polymorphisms (PCR-RFLP). Treatment response was evaluated by Response Evaluation Criteria in Solid Tumors (RECIST). Association of all clinical data (responses, recurrence and survival of patients) and single nucleotide polymorphisms (SNPs) was analysed by using SPSS (version 21.0).

**Results:** Patients with TA/AA genotype of *CYP2E1* T>A polymorphism showed significantly poor response while those with GC/CC genotype of *RAD51* G>C showed better response (*p* = 0.008, *p* = 0.014 respectively). Death was significantly higher in patients with GG genotypes of *RAD51* G>C and *XRCC1* G>A (*p* = 0.006, *p* = 0.002 respectively). Women with GC+CC genotype of *RAD51* G>C and AG+GG of *XRCC1* showed better survival and also reduced risk of death (HR = 0.489, *p* = 0.008; HR = 0.484, *p* = 0.003 respectively).

**Conclusion:** Results suggested that *CYP2E1* T>A (rs6413432), *RAD51* G>C (rs1801320), and *XRCC1* G>A (rs25487) polymorphisms may be used as predictive markers for clinical outcomes in cervical cancer patients undergoing cisplatin based concomitant chemoradiotherapy.

## Introduction

Cervical cancer is the most common cancer among women all over the world and majority of cases are diagnosed at advanced stage (1, 2). In early stage of cervical cancer, surgery and radiation therapy are equally effective, but for patients with advanced stage, chemoradiotherapy is the preferred mode of treatment (3). A varied outcome after chemoradiotherapy is observed in cervical cancer patients, these varied responses in individuals are often due to differences in their genetic constitution (4). Ionizing radiations induces DNA damage, including double strand DNA breaks (DSBs), Single-strand DNA breaks (SSBs) and DNA-DNA crosslinks while platinum compounds such as cisplatin forms cisplatin-DNA adducts (5, 6). These lesions are repaired by multiple repair pathways, homologous recombination (HR), non-homologous end-joining (NHEJ), base excision repair (BER), nucleotide excision repair (NER) and if repaired inadequately lead to cell death and increasing radiation sensitivity.

Genetic polymorphisms in *CYP* and DNA repair genes are associated with differential repair activities and may explain inter-individual differences in treatment response influencing clinical outcome (7–9). Polymorphisms in these DNA repair genes can modulate their total repair capacity as well as influence the removal of platinum-DNA adducts, persistence of which underpins the antitumor potential of chemoradiation (10, 11). RAD51 plays an important role in homologous recombination (HR) repair of DNA during DSBs damage caused by ionising radiation and alkylating agents (12). *RAD51* G172T polymorphism showed association with overall survival of cervical cancer patients treated with chemoradiotherapy (CRT) (2). XRCC1 plays critical role in both BER and single-stranded break repair (SSBR) processes and removes oxidative DNA damage caused by exposures to ionizing radiation or alkylating agents like cisplatin (13). Higher *XRCC1* expression is associated with poor response and survival, particularly in patients with head and neck squamous cell carcinoma (HNSCC) receiving chemoradiation (14). DNA repair gene polymorphisms, particularly *XRCC1* Arg399Gln, may modify the response to gemcitabine-platinum combination chemotherapy (15). The *XRCC3*-241Thr/Thr genotype was associated with adverse progression-free survival of colorectal cancer patients (16).

A clear understanding of the molecular genetics of cervical cancer is necessary to deduce new therapeutic strategies that will benefit patients suffering from the disease. In this study, we have investigated the role of different genetic polymorphisms *viz. CYP1A1* T>C (rs4646903), *CYP1A1* A>G (rs1048943), *CYP2E1* T>A (rs6413432), *RAD51* G>C (rs1801320), *XRCC1* G>A (rs25487), *XRCC2* G>A (rs3218536) and *XRCC3* C>T (rs861539) in the treatment outcome of cisplatin based chemoradiation in cervical cancer patients.

## Methods and Materials

The patients prescribed for cisplatin based concomitant chemoradiation (CRT) treatment were recruited for this study from the Departments of Radiotherapy and Obstetrics and Gynecology, King George’s Medical University (KGMU), Lucknow, India. The recruited women were with no associated co-morbid conditions and had received no previous radiation or chemotherapy. Demographic and clinical characteristics of patients were obtained from medical records while staging and clinical diagnosis of patients were performed by expert clinicians as per guidelines of International Federation of Gynecology and Obstetrics (FIGO) 2009. Samples were collected after informed consent of all study subjects and approval of Institutional Ethics Committee of KGMU, Lucknow (No.274/R.Cell-10). Five milliliters venous blood samples were obtained from 244 subjects for genotyping study at the start of treatment regimen.

Doses of both therapies (chemotherapy and radiation) were same for all patients. All patients received a total dose of 50 Gy in 25 fractions of pelvic external beam radiotherapy with weekly 40 mg/m^2^ concomitant cisplatin followed by three applications of high dose rate (HDR) intracavitory brachytherapy (7 Gy/fraction at 1-week interval). The patients who violated the treatment protocol or did not complete the planned chemoradiation dose were excluded from the study. The patient response to treatment was measured by response evaluation criteria in solid tumors (RECIST) version 1.0 after 1 month of treatment. Patients were followed-up after treatment and checked for survival. The primary endpoint was overall survival (OS) observed from the date of diagnosis to the date of death from any cause. Women who were alive at the end of the study were censored.

Genomic DNA was extracted from venous blood by salting out method with slight modification (17). DNA samples were checked by agarose gel electrophoresis. The *CYP1A1* T>C (rs4646903), *CYP1A1* A>G (rs1048943), *CYP2E1* T>A (rs6413432), *RAD51* G>C (rs1801320), *XRCC1* G>A (rs25487), *XRCC2* G>A (rs3218536) and *XRCC3* C>T (rs861539) polymorphisms were genotyped by Restriction Fragment Length Polymorphism (PCR-RFLP) by using specific primers (F-5′ACTCACCCTGAACCCCATTC3′, R-5′GGCCCCAACTACTCAGAGGCT3′; F-5′CTGTCTCCCTCTGGTTACAGGAAGC3′, R-5′TTCCACCCGTTGCAGCAGGATAGCC3′; F-5′TCGTCAGTTCCTGAAAGCAGG3′, R-5′GAGCTCTGATGGAAGTATCGCA-3′; F-5′TGGGAACTGCAACTCATCTGG3′, R-5′GCGCTCCTCTCTCCAGCAG3′; F-5′TTGTGCTTTCTCTGTGTCCA3′, R-5′TCCTCCAGCCTTTTCTGATA3′; F-5′TGTAGTCACCCATCTCTCTGC3′, R-5′AGTTGCTGCCATGCCTTACA3′; and F-5′GGTCGAGTGACAGTCCAAAC3′, R-5′CTACCCGCAGGAGCCGGAGG3′, respectively) and specific restriction enzymes (*MspI*, *BsrDI*, *DraI*, *BstNI*, *MspI*, *HphI* and *NlaIII).*

Demographic and clinical information was correlated with genotypes using *χ* ^2^ analysis and Fisher’s exact test (for categoric variables) and one-way analysis of variance (for continuous variables). Genotype and overall survivals were evaluated by Kaplan-Meier function and Cox proportional hazards model. Log-rank test was used to detect differences in overall survival across different genotypes. 95% Confidence Intervals (CIs) and Hazard ratios (HRs) were estimated using multivariate Cox proportional hazards model/Cox regression analysis. The regression data was adjusted for age, stage and histopathology. All differences in *p* values were considered statistically significant for *p* < .05. All statistical analyses were performed by SPSS (Version 21.0).

## Results

Out of 244 cervical cancer patients, 227 were included in the study. Seventeen cases were excluded due to protocol violations. Mean age of patients was 49.0 ± 8.68 years. Histopathologically, 216 patients (95.2%) had squamous cell carcinoma and remaining 11 (4.8%) had adenocarcinoma. Squamous cell carcinoma was distributed into three categories: 96 well (44.4%), 79 moderate (36.6%) and 21 poor (9.7%) while no differentiation was reported in 20 cases (9.3%). The staging of tumor according to FIGO was 117 cases (51.5%) with stage IIB and 110 (48.5%) with stage IIIA+IIIB (Table 2). Response Evaluation Criteria in Solid Tumors (RECIST) was used to assess treatment response as complete response (CR), partial response (PR), stable disease (SD) and progressive disease (PD). CR and PR were considered as responders while SD and PD as non-responders.

The distribution of *CYP1A1* T>C rs4646903 genotypes showed 33.5% cases with TT and 66.5% with TC/CC while distribution of *CYP1A1* A>G rs1048943 showed 54.6% cases with AA and 45.4% with AG/GG. The distribution of *CYP2E1* T>A rs6413432 genotypes showed 81.1% cases with TT, 18.9% with TA/AA. The distribution of *RAD51* G>C rs1801320 genotypes showed 58.6% cases with GG and 41.4% with GC/CC while distribution of *XRCC1* G>A rs3218536 showed 42.7% cases with GG and 57.3% with GA/AA. The *XRCC2* G>A rs3218536 genotypes showed 78.9% cases with GG, 21.1% with GA/AA. However, distribution of *XRCC3* C>T rs861539 showed 59.5% cases with CC genotypes and 40.5% with CT/TT genotypes. The significant differences in treatment response were found for *CYP2E1* T>A rs6413432*,* but not for *CYP1A1* T>C rs4646903 and A>G rs1048943 polymorphisms. The cases with TA genotype of *CYP2E1* T>A rs6413432 polymorphism showed significant decrease in treatment response when compared to those with TT genotype (36.4 vs 13.4%, *p* = .002). In case of *RAD51* G>C rs1801320, homozygous GG genotype had a significantly bad response when compared with GC genotype (55.2 vs 78.8%; *p* = .011) and same results were found with CC genotype but no statistical significance (5.7 vs 6.1%; *p* = .717). In case of *XRCC1* G>A rs25487, *XRCC2* G>A rs3218536 and *XRCC3* C>T rs861539, we did not observe any significant association (*p* > .05, Table 1).

**TABLE 1:** Association of genotypes of *CYP1A1* T>C rs4646903, *CYP1A1* A>G rs1048943, *CYP2E1* T>A rs6413432, *RAD51* G>C rs1801320, *XRCC1* G>A, rs25487 *XRCC2* G>A rs3218536 and *XRCC3* C>T rs861539 polymorphisms with clinical response of cervical cancer cases (*n* = 227).

Genotypes	Clinical Response			OR (95% CI)	*p* value
	N	CR+PR, (%)	SD+PD, (%)		
	227	194 (85.5)	33 (14.5)		
CYP1A1 rs4646903T>C					
TT	76	68 (35.1)	8 (24.2)	1.0 (Ref)	
TC	113	93 (47.9)	20 (60.6)	0.182 (0.760–4.396)	.178
CC	38	33 (17.0)	5 (15.2)	1.30 (0.391–4.243)	.677
TC/CC	151	126 (64.9)	25 (75.8)	1.70 (0.722–3.942)	.228
CYP1A1 rs1048943A>G					
AA	124	105 (54.1)	19 (57.6)	1.0 (Ref)	
AG	97	83 (42.8)	14 (42.4)	0.93 (0.441–1.969)	.854
GG	6	6 (3.1)	0 (0.0)	0	0
AG/GG	103	89 (45.9)	14 (42.4)	0.90 (0.412–1.833)	.713
CYP2E1 rs6413432T>A					
TT	184	163 (84.0)	21 (63.6)	1.0 (Ref)	
TA	38	26 (13.4)	12 (36.4)	3.58 (1.576–8.144)	.002
AA	5	5 (2.6)	0	0	0
TA/AA	43	31 (16.0)	12 (36.4)	3.00 (1.341–6.731)	.008
RAD51 rs1801320G>C					
GG	133	107 (55.1)	26 (78.8)	1.0 (Ref)	
GC	81	76 (39.2)	5 (15.2)	0.27 (0.099–0.737)	.011
CC	13	11 (5.7)	2 (6.1)	0.75 (0.156–3.584)	.717
GC/CC	94	87 (44.8)	7 (21.2)	0.33 (0.137–0.799)	.014
XRCC1 rs25487G>A					
GG	97	80 (42.2)	17 (51.5)	1.0 (Ref)	
GA	98	85 (43.8)	13 (39.4)	0.72 (0.329–1.576)	.411
AA	32	29 (14.9)	3 (9.1)	0.49 (0.133–1.784)	.277
GA/AA	130	114 (58.8)	16 (48.5)	0.66 (0.315–1.384)	.272
XRCC2 rs3218536G>A					
GG	179	153 (78.9)	26 (78.8)	1.0 (Ref)	
GA	46	40 (20.6)	6 (18.2)	0.90 (0.340–2.290)	.798
AA	2	1 (0.5)	1 (3.0)	5.90 (0.357–97.043)	.215
GA+AA	48	41 (21.1)	7 (21.2)	1.01 (0.407–2.478)	.992
XRCC3 rs861539C>T					
CC	135	113 (58.2)	22 (66.7)	1.0 (Ref)	
CT	78	68 (35.1)	10 (30.3)	0.76 (0.337–1.691)	.495
TT	14	13 (6.7)	1 (3.0)	0.40 (0.049–3.177)	.383
CT/TT	92	81 (41.8)	11 (33.3)	0.70 (0.320–1.519)	.364

CI, confidence interval; OR, odds ratio; significant association (*p* < 0.05); CR, complete response; PR, partial response; SD, stable disease; PD, progressive disease; CR+PR, Responders; SD+PD, Non-responder; 1.0 (Reference).

**FIGURE 1:**
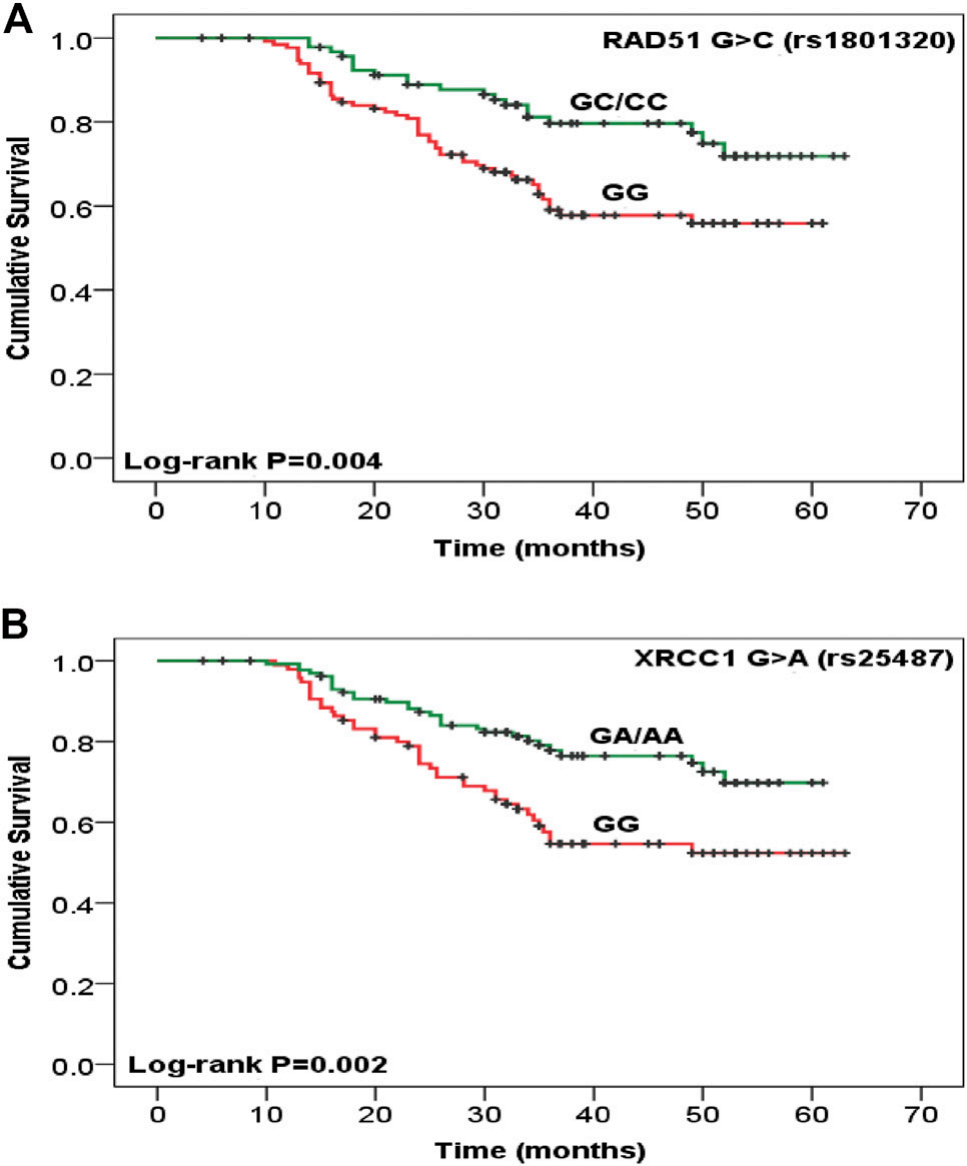
Kaplan-Meier function for overall survival after chemoradiotherapy treatment among cervical cancer patients with **(A)** *RAD51* G>C rs1801320, GG vs. GC/CC (*n* = 133 vs. 94) and **(B)** *XRCC1* G>A rs25487, GG vs. GA/AA (*n* = 97 vs. 130) genotypes. Test for survival difference by log- rank methods.

Disease recurrence was significantly decreased in women with TA/AA genotypes of *CYP2E1* T>A rs6413432 (*p* = .016) but *CYP1A1* T>C rs4646903 and A>G rs1048943 polymorphisms did not show any association (Table 2). Most of the cases with GC+CC genotypes of *RAD51* G>C rs1801320 and GA/AA genotypes of *XRCC1* G>A rs25487 were found to be alive at the end of study period (*p* = .006 and *p* = .002, respectively) while none with *XRCC2* G>A rs3218536 and *XRCC3* C>T rs861539 genotypes exhibited similar response. The risk of recurrence was significantly reduced in GA/AA genotypes of *XRCC1* G>A rs25487 (*p* = .025) (Table 2). The median follow-up duration for all cases was 34 months (range, 4.2–63.0 months). During the study period (2009–2011), 31.3% cases succumbed to death. Association of genotypes with overall survival as analysed by Cox proportional hazards model (HR), adjusted for age, stage and histopathology is shown in Table 3. There was significant reduction in hazard of death (HR = 0.489) among women with GC/CC genotypes of *RAD51* G>C rs1801320 when compared with women having GG genotype and HR = 0.484 with GA/AA genotypes of *XRCC1* G>A rs25487 when compared with women having AA genotype (*p* = .008 and *p* = .003 respectively) (Table 3). The Kaplain-Meier function and Log rank test for survival in cases with genotypes are shown in Figures 1A,B. There was no association with the *CYP1A1* A>G rs1048943 and *CYP2E1* T>A rs6413432 genotypes. The GC+CC genotype of *RAD51* G>C rs1801320 and GA/AA genotype of *XRCC1* G>A rs25487 were associated with better overall survival (log-rank, *p* = .004 and *p* = .002 respectively) (Figures 1A,B). There was no association with *XRCC2* G>A rs3218536 and *XRCC3* C>T rs861539 polymorphisms.

**TABLE 2:** Association of genotypes of *CYP1A1* T>C rs4646903, *CYP1A1* A>G rs1048943, *CYP2E1* T>A rs6413432, *RAD51* G>C rs1801320, *XRCC1* G>A, rs25487 *XRCC2* G>A rs3218536 and *XRCC3* C>T rs861539 polymorphisms with vital status and recurrence of cervical cancer cases (*n* = 227).

Genotypes	Vital Status				Recurrence		
	Cases	Alive	Deceased	*p* value	Disease Free	Never Disease Free/Recurred	*p* value
	*n* = 227	*n* = 156	*n* = 71		*n* = 160	*n* = 67	
CYP1A1 T>C rs4646903							
TT	76 (33.5)	49 (31.4)	27 (38.0)	.327	48 (30.0)	28 (41.8)	.086
TC/CC	151 (66.5)	107 (68.6)	44 (62.0)		112 (70.0)	39 (58.2)	
CYP1A1 A>G rs1048943							
AA	124 (54.6)	88 (56.4)	36 (50.7)	.423	83 (51.9)	41 (61.2)	.198
AG/GG	103 (45.4)	68 (43.6)	35 (49.3)		77 (48.1)	26 (38.8)	
CYP2E1 T>A rs6413432							
TT	184 (81.1)	125 (80.1)	59 (83.1)	.596	133 (83.1)	51 (76.1)	.219
TA/AA	43 (18.9)	31 (19.9)	12 (16.9)		27 (16.9)	16 (23.8)	
RAD51 G>C rs1801320							
GG	133 (58.6)	82 (52.6)	51 (71.8)	.006	88 (55.0)	45 (67.2)	.09
GC/CC	94 (41.4)	74 (47.4)	20 (28.2)		72 (45.0)	22 (32.8)	
XRCC1 G>A rs25487							
GG	97 (42.7)	56 (35.9)	41 (57.7)	.002	65 (40.6)	32 (47.8)	.322
GA/AA	130 (57.3)	100 (64.1)	30 (42.3)		95 (59.4)	35 (52.2)	
XRCC2 G>A rs3218536							
GG	179 (78.9)	124 (79.5)	55 (77.5)	.729	126 (78.8)	53 (79.1)	.952
GA/AA	48 (21.1)	32 (20.5)	16 (22.5)		34 (21.2)	14 (20.9)	
XRCC3 C>T rs861539							
CC	135 (59.5)	95 (60.9)	40 (56.3)	.517	89 (55.6)	46 (68.7)	.068
CT/TT	92 (40.5)	61 (39.1)	31 (43.7)		71 (44.4)	21(31.3)	

Significant association (*p* < .05).

**TABLE 3:** Association of genotypes of *CYP1A1* T>C rs4646903, *CYP1A1* A>G rs1048943, *CYP2E1* T>A rs6413432, *RAD51* G>C rs1801320, *XRCC1* G>A rs25487, *XRCC2* G>A rs3218536 and *XRCC3* C>T rs861539 polymorphisms and survival after treatment (CRT) for cervical cancer.

Genotypes	Alive cases, (%)	Death cases, (%)	HR^a^ (95% CI)	*p* value
	156	71		
CYP1A1 rs4646903T>C				
TT	49 (31.4)	27 (38.0)	1.0 (Ref)	.196
TC/CC	107 (68.6)	44 (62.0)	0.724 (0.443–1.82)	
CYP1A1 rs1048943A>G				
AA	88 (56.4)	36 (50.7)	1.0 (Ref.)	.463
AG/GG	68 (43.6)	35 (49.3)	1.295 (0.743–1.921)	
CYP2E1 rs6413432T>A				
TT	125 (80.1)	59 (83.1)	1.0 (Ref.)	.54
TA/AA	31 (19.9)	12 (16.9)	0.823 (0.441–1.536)	
RAD51 rs1801320G>C				
GG	82 (52.6)	51 (71.8)	1.0 (Ref)	.008
GC/CC	74 (47.4)	20 (28.2)	0.489 (0.287–0.832)	
XRCC1 rs25487G>A				
GG	56 (35.9)	41 (57.7)	1.0 (Ref.)	.003
GA/AA	100 (64.1)	30 (42.3)	0.484 (0.302–0.775)	
XRCC2 rs3218536G>A				
GG	124 (79.5)	55 (77.5)	1.0 (Ref.)	.465
GA/AA	32 (20.5)	16 (22.5)	1.233 (0.703–2.165)	
XRCC3 rs861539C>T				
CC	95 (60.9)	40 (56.3)	1.0 (Ref.)	.332
CT/TT	61 (39.1)	31 (43.7)	1.265 (0.787–2.032)	

CI, Confidence interval; HR, Hazard ratio; Significant association (*p* < .05); 1.0 (Reference).

a Adjusted for age, stage and histopathology.

## Discussion

Cisplatin based concomitant chemoradiation is the standard treatment of locally advanced cervical cancer (2). However, primary or acquired chemoradioresistance is a serious clinical problem that contributes to disease recurrence, progression and mortality (18). Poor response and high inter-individual variations in treatment response occurs among patients. Local and distant metastasis occurs due to the survival of some tumor cells leading to treatment failure. Therefore, new and more effective approaches are required to tackle this issue. The mechanisms of these heterogeneous responses to treatment are multifactorial and involve variability in genetic constitution (19).

Members of Xenobiotic metabolizing enzymes (XMEs) are CYP1A1, CYP1B1, CYP2D6, CYP2E1, mEH, NAT1 *etc,* which demonstrate their anti-neoplastic effects by producing reactive oxygen species (ROS) whose cytotoxic effects cause tumor cell death and are likely to impact the treatment efficacy as well as survival after treatment (20, 21). CYP1A1 and CYP2E1 are the important members of XME family and have been extensively studied as biomarkers for cancer risk prediction. Significant association of *CYP1A1* m2 (rs1048943) polymorphism with platinum drugs (e.g. Cisplatin) was observed, which represent an important class of anticancer agents and are frequently used in treatment of various types of solid cancers including cervical cancer (22, 23). In the present study, cases with homozygous TT/AA genotype of *CYP2E1* T>A rs7632 polymorphism showed poor treatment response as compared to heterozygous TT genotype with significant association (*p* = .002, Table 3). No association of *CYP1A1* (*CYP1A1* rs4646903, *CYP1A1* rs1048943 and *CYP2E1* T>A rs7632) polymorphisms was found with survival of cervical cancer patients treated with cisplatin based chemoradiation.

Radiotherapy and chemotherapy destroy cancer cells by inducing DNA damage. So, the treatment outcome may be dependent on DNA repair systems (24). It is known that ionizing radiation (IR) can damage DNA, producing single and double-strand breaks on DNA, as well as an indirect effect by generating reactive oxygen species (ROSs) in the cells (25). The DNA repair capacity of individuals consists of several pathways: nucleotide and base excision repair (BER), homologous recombination (HR), end joining, and telomere metabolism. BER of single-strand breaks and homologous repair of double-strand breaks (DSBs) are considered the most important pathways in repair of radiation-induced DNA damage (26). Many studies confirmed that genetic polymorphisms in DNA repair genes are associated with differential treatment outcomes of Chemoradiotherapy (9). Human RAD51 is required for meiotic/mitotic recombination and plays an important role in homology-dependent recombinational repair of DSBs, caused by ionizing radiation and alkylating agents (12). *RAD172* G>T (rs1801321) polymorphism has been associated with altered gene transcription (27). *RAD51* expression is an independent predictor for tumor progression as well as tumor recurrence (2). In the present study, response of treatment was significantly higher (*p* < .05) in individuals with “C” allele of *RAD51* G>C rs1801320 polymorphism (Table 1). A reduced hazard of death and better overall survival was observed among CRT treated women with GC/CC genotypes of *RAD51* G>C rs1801320 (*p* = .008, *p* = .004 respectively) (Figure 1A; Table 3). The individuals with GC/CC genotypes of *RAD51* G>C rs1801320 were alive for a longer period of time as compared to those with GG genotype at the end of study period (*p* = .006) (Table 2). X-Ray repair cross complementing group 1 (XRCC1) is a base excision repair (BER) protein that plays an important role in single-strand breaks repair (SSBR), DSBs repair, BER and following exposure to endogenous reactive oxygen species. XRCC1 deficiency results in hypersensitivity to chemoradiation. *XRCC1* R399Q polymorphism is a well-studied single nucleotide polymorphism (SNP) located in the BRCT1 domain and have been found to influence treatment response (25, 28). Two survival studies in ovarian cancer were conducted in Korea and Russia where no significant association was found with *XRCC1* +399 G>A (rs25487) polymorphism (29, 30). According to Miao et al. (31), individuals with AA genotype of *XRCC1* +399G>A polymorphism had a significant risk of death from ovarian cancer. Other studies have shown association of *XRCC1* with survival and/or risk in non-small-cell lung cancer, colorectal and laryngeal squamous cell cancer (9, 32). According to our results, occurrence of death was significantly reduced among patients having GA/AA genotypes of *XRCC1* +399 G>A (*p* = 0.002). Most of the cases with GA/AA genotype of *XRCC1* +399G>A were found to be alive at the end of the study period as compared to cases with AA genotype (*p* = .003) (Figure 1B). XRCC2 is one of the main components of RAD51-related protein family required for correct chromosome segregation and apoptotic response to DSBs (33). The G>A (rs3218536) polymorphism of *XRCC2* was an independent prognostic factor for overall survival in non-small cell lung cancer (NSCLC) cases treated with definitive radiotherapy (34). In pancreatic cancer, the AA genotype of *XRCC2* G>A rs3218536 was associated with a significantly shorter survival than the GG/GA genotype (35). High expression of XRCC3 in esophageal squamous cell carcinoma (ESCC) is associated with chemoradiotherapy resistance and predicts poor survival in patients (36). According to Bewick et al (2006) survival of breast cancer patients was higher in women having *XRCC3* C>T rs18067 polymorphism (37). In present study, no association of *XRCC2* rs3218536G>A and *XRCC3* rs861539C>T polymorphisms was observed with treatment outcome in cervical cancer patients.

In this study, we have focused on the effect of genetics on inter-individual differences in response to DNA damaging agents. The differential activity of cytochrome P-450 and DNA repair enzymes have an impact on treatment outcome of individual patients. Therefore, this kind of study may help clinicians to alter treatment strategies for cervical cancer patients on a personalized basis.

## Summary Table

### What is Known About This Subject

• Cisplatin based concomitant chemoradiation is the standard treatment for locally advanced cervical cancer. Poor response and high inter-individual variations in treatment response occurs due to differences in genetic makeup.
• The differential activity of metabolic (e.g., cytochrome P-450) and DNA repair enzymes have an impact on treatment outcome.
• Genetic polymorphisms in metabolic and DNA-repair enzymes contribute to inter-patient variability in treatment response.

### What This Paper Adds

• Genetic variants in drug metabolizing and DNA repair genes with treatment outcome of CRT in cervical cancer patients.
• *CYP2E1*rs6413432, *RAD51*rs1801320, and *XRCC1*rs25487 were associated with inter-individual variations in response to chemoradiotion.
• These polymorphisms may act as prognostic biomarkers for prediction of clinical outcome of CRT in cervical cancer patients.

## Data Availability

The original contributions presented in the study are included in the article, further inquiries can be directed to the corresponding author.
